# Different methods of determining centric relation – comparison with a digital mandibular motion analyser

**DOI:** 10.1186/s12903-024-04131-x

**Published:** 2024-03-18

**Authors:** Bálint Jász, Szilvia Ambrus, Tamás Garay, Péter Schmidt, Péter Hermann, Szandra Körmendi, Máté Jász

**Affiliations:** 1 Department of Prosthodontics, Faculty of Dentistry, Semmelweis University, Szentkirályi u. 47, Budapest, 1088 Hungary; 2 Faculty of Information Technology and Bionics, Pázmány Péter Catholic University, Budapest, Hungary; 3 Division of Oncology, Department of Internal Medicine and Oncology, Faculty of Medicine, Semmelweis University, Budapest, Hungary

**Keywords:** Centric relation, Gothic arch tracing, Dawson’s method, Digital motion analysis

## Abstract

**Background:**

Finding and registering the maxillary–mandibular jaw relation is crucial in dental practice. Several comparative studies have been conducted to investigate the reproducibility and accuracy of techniques for determining the centric relation (CR) position of the mandible. The aim of our study was to determine which of seven different CR determination methods had the smallest deviation from the theoretical zero with the help of a digital mandibular motion analyser. The chosen theoretical zero position, the maximal intercuspal position (MIP), is the most reproducible and widely used position.

**Methods:**

Thirty-four volunteers (24 females and 10 males) with a mean (SD) age of 29.1 (± 7.3) years with a negative history of temporomandibular disorder (TMD) participated in the study. A digital mandibular motion analyser was used to register the condylar position after the use of each technique for the determination of CR. The calibration was performed to the maximal intercuspal position (MIP) for each volunteer. The investigated techniques were (A) the gothic arch tracer, (B) the adduction field method, (C) Dawson’s bimanual manipulation, (D) the patient placing the tongue tip on the palatal rugae, (E) the patient placing the tongue tip to the border of the hard and soft palate, (F) the patient actively pulling the chin backwards, and (G) the examiner pushing the patient’s chin back.

**Results:**

The position of the mandibular condyle was illustrated in a three-dimensional coordinate system, where the origin represented the MIP. Among the seven methods examined, five showed significant deviations compared to the MIP. Among these, two methods resulted in posterior deviation of the condyles. Methods C and E coincided with the MIP in all directions.

**Conclusions:**

Within the limitations of our study, we found that the smallest deviations from our theoretical zero (MIP) among the investigated centric relation determining methods were obtained with the bimanual mandibular manipulation technique derived from Dawson and the placement of the tongue tip on the border of the hard and soft palate (linguomandibular homotrophy theory).

## Background

The term centric relation (CR), which describes the relationship between the mandibular condyle and the articular fossa, has undergone significant changes in recent decades. While the first to fourth editions (1977) of the Glossary of Prosthodontic Terms (GPT) defined the most posterior position of the condyle as the CR, from the fifth edition (1987) to the latest ninth edition (2017), the anterior-superior position has been indicated as the centric position [1–4]. This repeatable maxillomandibular relationship is used in various fields of dentistry, including extensive prosthodontic rehabilitation, temporomandibular disorders (TMD) and orthodontic treatment [5–7]. As a result, numerous methods for determining the CR position of the mandible have been developed since the 1950s [8]. Several comparative studies have been conducted to determine the most appropriate technique [9–11]. Not only the definition but also the role of CR has changed and continues to change. In recent years, in addition to the CR, the importance of the MIP has become increasingly prominent, as has the need to maintain the original jaw relationship for as long as possible [7, 12].

Some studies have focused on the reproducibility of the methods, while others have focused on their accuracy. Accuracy was defined as the determination of the location of the condyle as close as possible to the current CR position. The majority of these studies use orthopantomograms, cone beam computed tomography (CBCT) or magnetic resonance imaging (MRI) examinations of the temporomandibular joint or traditional (average value) articulators [9, 13–16]. By involving digital technology, new opportunities for the comparative analysis of techniques for reproducing the CR position have opened up. In our study, we used a digital mandibular motion analyser (KaVo Arcus Digma 2). A recent review revealed ultrasound motion analyser devices to be sufficiently accurate. The device used in this study is one of these instruments [17].

In comparative studies of procedures for determining the CR position, there should be a distinction between studies performed on edentulous patients and patients with a stable maximum intercuspal position (MIP) [7, 12]. Patients with a stable MIP were recruited for this study. The study aimed to determine the extent to which the condylar positions obtained through different methods deviated from the MIP along the three axes. The null hypothesis was that none of the condyle positions determined by the different methods are different from the reference position.

## Methods

The study was conducted at the Department of Prosthodontics of Semmelweis University (Budapest, Hungary). Ethical approval was granted by the Semmelweis University Regional and Institutional Committee of Science and Research Ethics (No. 92/2013). The study was performed between 2014 and 2018. There were 34 volunteers who participated in the study, including 24 females and 10 males. The study group was selected from the staff and students of the Faculty of Dentistry of Semmelweis University. The mean (SD) age of the participants was 29.1 (± 7.3) years.

Before conducting the measurements, general and dental anamneses were taken. The selection criteria for the study were as follows: (1) general good health and absence of dental and jaw developmental disorders; (2) preserved or restored dentition (excluding wisdom teeth); (3) in patients with restored dentition, the occluding surfaces of the restoration are not guiding surfaces in the articulatory movements; (4) no previous orthodontic treatment; (5) no medical history of temporomandibular disorders (TMD) and no complaints of TMD at the time of the examination; and (6) no occurrence of bruxism or other parafunctions and no abnormal tooth guidance (e.g., hyperbalanced contacts) based on the patient’s history and dental examination. Dental status examination was performed together with patient examination according to the DC-TMD protocol to identify eventual temporomandibular joint (TMJ) and muscle-related complaints. The chewing system was assessed by an examiner (MD, DMD) who is experienced in DC-TMD examination and has worked in the field of gnathology for twenty years.

**Fig. 1:**
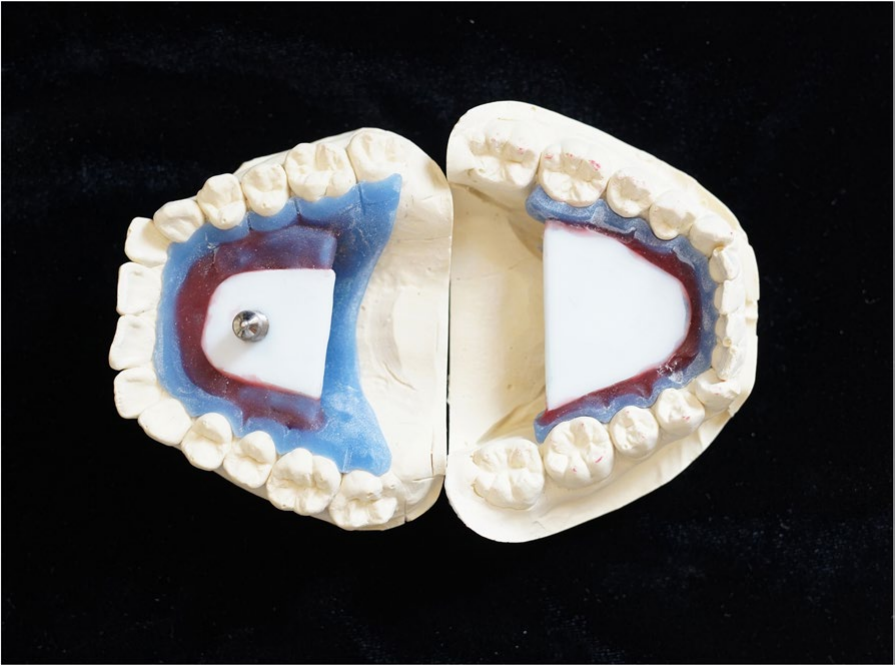
Gothic arch tracer

In the first session, putty-wash impressions of the lower and upper jaws were taken (Zetaplus, Oranwash, Zhermack, Badia Polesine, Italy). The impressions were poured with type IV die stone (Fuji Rock, GC International AG, Luzern, Switzerland) in the dental laboratory. From the completed casts, an intraoral gothic arch tracer was produced (Fig. 1). The pin was positioned on the upper jaw, while the tracing table was positioned on the lower jaw. The tracing table was placed parallel to the occlusal plane.

 In the second session, the measurements were performed by the same examiner each time. The examinations were conducted using a KaVo Arcus Digma 2 digital motion analyser (KaVo Gmbh, Biberach, Germany). In the initial step, a paraocclusal clutch was fixed on the vestibular surface of the mandibular teeth using a cold polymerizing composite resin material (Structure 2 SC, Voco GmbH, Cuxhaven, Germany). The ultrasound transmitter was attached to the clutch with magnets. Subsequently, the facebow was positioned using the left infraorbital point as a reference, so the reference plane for the examination was the Frankfort horizontal plane. Before starting the digital analysis, an arbitrary axis calibration was performed: the position of the mandible transmitter was adjusted to the Frankfort horizontal plane with a calibration pin of known length. To determine the intercondylar axis, the lateral poles of the condyles were zeroed as well.

During the examination, the electronic position analysis (EPA) module was used, and calibration was performed to the maximum intercuspal position (MIP) of the mandible. The examiner determined the CR using seven different methods and registered the position of the mandible relative to the maxilla, along with the corresponding condylar position, after each determination.

The following CR determination techniques were investigated: (A) a gothic arch tracer, (B) the adduction field method [18] (in both methods, registration was performed at a slightly open position (at an elevated occlusal vertical dimension) due to the characteristics of the gothic arch tracer; the smallest possible increase was achieved with this intraoral drawing device), (C) Dawson’s bimanual manipulation [19], (D) having the patient place the tip of the tongue on the palate in the area of the palatal rugae and close their mouth until the first contact of teeth occurred [20], (E) having the patient withdraw the tip of the tongue back to the border of the hard and soft palate and close their mouth until the first contact of teeth occurred (methods (D) and (E) are based on the theory of linguomandibular homotrophy) [20], (F) having the patient close the jaw until the first contact of teeth occurred while actively retruding the chin, and (G) the examiner pushing the patient’s chin back during closing with a force of 20 N and fixing the final position to ensure that the condyle was not in a forced posterior position during the previous part of the examination [21]. All of the examinations were performed by the same examiner, who graduated with MD and DMD degrees and worked in the field of gnathology for twenty years. Two randomly selected patients were re-examined. The second measurements were duplicates and were not included in the final statistical analysis. The weighted kappa coefficients for intraexaminer reliability ranged from 0.88 to 0.93.

Condylar positions corresponding to each determined jaw relationship were exported by using the manufacturer’s program (KaVo KiD, KaVo Gmbh, Biberach, Germany). Each condylar position was displayed in a three-dimensional coordinate system, where the origin was set as the initially calibrated MIP. On the sagittal x-axis, a positive value indicated a forwards/anterior position from the MIP, and a negative value indicated a more posterior/backwards position. Along the vertical y-axis, a positive value indicated a more cranial position than the MIP, while a negative value represented a more caudal position. For the horizontal z-axis, a positive deflection represented a deviation to the left, while a negative deflection represented a deviation to the right. Statistical differences were evaluated through the computation of the mean and 95% confidence interval (CI), signifying that upon replicating our measurements using identical sample sizes and methodologies, the mean outcomes would fall within the indicated interval with a probability of 95% (corresponding to a significance level of 0.05). Exported data were processed using GraphPad Prism software (GraphPad Software, Boston, USA) for statistical analysis.

## Results

**Fig. 2:**
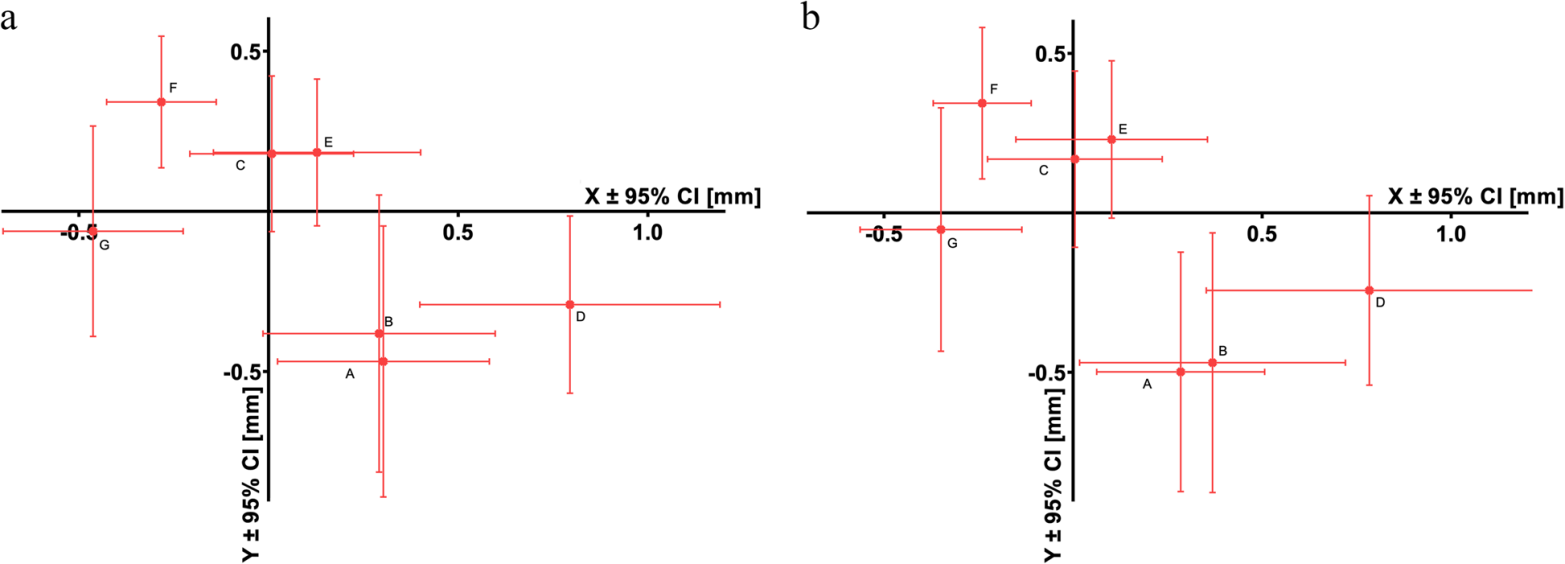
Deviations of the condyle from the MIP (origin) after different CR determination techniques on the left (**a**) and right (**b**) sides: apex of the gothic arch tracer (A), adduction field (B), Dawson technique (C), tongue at the palatal rugae (D), tongue at the hard-soft palate border (E), active retrusion (F), and passive retrusion (G)

After the EPA examination, the positions of both the right and left condyles were represented in a three-dimensional coordinate system. Therefore, for each determination method, one point was marked in the coordinate system for each condyle. The data were evaluated along the axes of the coordinate system. The deviations from the origin (MIP) were indicated along the x-, y- and z-axes for each method with a 95% confidence interval. Along the transverse z-axis, the deviation between the individual positions was 0 mm, with a 95% CI [-0.03, 0.03]. Thus, these values can be considered negligible in the study. Therefore, the distance between the MIP and the measuring point was calculated as a common vector between the x-axis and y-axis (Fig. 2).

On the x-axis, the positions determined by the Dawson technique (C) and by placing the tongue on the border of the hard and soft palate (E) on both sides and the adduction field method (B) on the left side did not differ significantly from those of the MIP. However, there was a significant difference between the MIP and the positions determined by the apex of the gothic arch tracer (A), by placing the tongue on the palatal rugae (D), and by the active and passive retrusion of the chin (F and G) on both sides and by the adduction field method (B) on the right side.

On the y-axis, the positions determined by the Dawson manipulation (C), placing the tongue to the border of the hard and soft palate (E), and mandibular retrusion by the operator (G) did not significantly differ from the MIP (y = 0) on either side. The positions determined by placing the tongue to the rugae (D) on the right side and by the adduction field method (B) on the left side also did not significantly differ from the MIP. However, there was a significant difference from the MIP in the positions determined by the apex of the gothic arch tracer (A) and by patient retrusion of the mandible (F) on both sides. Placing the tongue to the rugae (D) on the left side and the adduction field method (B) on the right side also yielded significant differences from the MIP (Tables 1 and 2).

**Table 1:** Raw deviations (averages and 95% confidence intervals) from the MIP on the x (sagittal) and y (vertical) axes for different CR determination techniques on the right side. Significant differences from the MIP (i.e., sagittal = 0, vertical = 0) are marked with italics and asterisks

	Sagittal	Vertical
Apex of the gothic arch tracer (A)	0.28 mm, 95% CI [0.06, 0.51]*	-0.5 mm, 95% CI [-0.87, -0.12]*
Adduction field (B)	0.37 mm, 95% CI [0.02, 0.72]*	-0.47 mm, 95% CI [-0.88, -0.06]*
Dawson technique (C)	0 mm, 95% CI [-0.23, 0.23]	0.17 mm, 95% CI [-0.11, 0.45]
Tongue at the palatal rugae (D)	0.78 mm, 95% CI [0.35, 1.22]*	-0.24 mm, 95% CI [-0.54, 0.05]
Tongue at the hard-soft palate border (E)	0.1 mm, 95% CI[-0.15, 0.36]	0.23 mm, 95% CI [-0.02, 0.48]
Active retrusion (F)	-0.24 mm, 95% CI [-0.37, -0.11]*	0.34 mm, 95% CI [0.11, 0.58]*
Passive retrusion (G)	-0.35 mm, 95% CI [-0.56, 0.14]*	-0.05 mm, 95% CI [-0.43, 0.33]

**Table 2:** Raw deviations (averages and 95% confidence intervals) from the MIP on the x (sagittal) and y (vertical) axes for different CR determination techniques on the left side. Significant differences from the MIP (i.e., sagittal = 0, vertical = 0) are marked with italics and asterisks

	Sagittal	Vertical
Apex of the gothic arch tracer (A)	0.3 mm, 95% CI [0.02, 0.58]*	-0.47 mm, 95% CI [-0.89, -0.05]*
Adduction field (B)	0.29 mm, 95% CI [-0.01, 0.6]	-0.38 mm, 95% CI [-0.81, 0.05]
Dawson technique (C)	0.01 mm, 95% CI [-0.21, 0.22]	0.18 mm, 95% CI [-0.06, 0.42]
Tongue at the palatal rugae (D)	0.79 mm, 95% CI [0.4, 1.19]*	-0.29 mm, 95% CI [-0.57, -0.01] *
Tongue at the hard-soft palate border (E)	0.13 mm, 95% CI [-0.15, 0.4]	0.18 mm, 95% CI [-0.05, 0.41]
Active retrusion (F)	-0.28 mm, 95% CI [-0.43, -0.14]*	0.34 mm, 95% CI [0.14, 0.55]*
Passive retrusion (G)	-0.46 mm, 95% CI [-0.7, -0.23]*	-0.06 mm, 95% CI [-0.39, 0.27]

## Discussion

The centric relation of the mandible is still a controversial topic. Some authors consider a discrepancy between the CR and the MIP (centric slide) to be a predisposing factor for TMD [22, 23]. Considering the very frequent prevalence of centric slides, others do not consider it a pathological condition and thus do not consider the role of CR to be central [7, 12]. These authors argue that, in principle, the MIP is the ideal position, independent of the condylar position. Achieving the CR position is only required in cases of restorations for partially or completely edentulous patients, full-mouth rehabilitation, and orthodontic and orthognathic treatments involving the whole jaw [12]. However, numerous studies have compared different CR determination methods to each other or to a preferred position (mostly the MIP) [9, 24–28]. In most cases, dentate patients formed the study group, but in some cases, edentulous patients were also involved [9, 26, 29]. The main differences among these studies are in the examination procedures used. The oldest and most widely used method is to mount the casts in an articulator after determining the CR position by each method and then compare them [16, 24, 25]. The advantages of this method are its simplicity and relatively low cost, but its limitations are the inaccuracy of mounting the casts and the millimetre scale used for measurement. In some studies, different imaging technologies have been used to examine the different positions [13, 14, 21]. With these methods, accurate images of the condyle-fossa relationship can be obtained, but this technique is relatively expensive, and when using CBCT, radiation exposure is not negligible.

In addition to these previous studies, the use of digital technology represents a new methodology. Axiography is an examination method that has been used for almost 100 years to assess the position and movements of the lower jaw relative to a reference plane. Axiography has improved with the use of digital technology and digital motion analysis [30]. In our study, we used an Arcus Digma 2, a digital motion analyser that operates with ultrasound. The device includes four ultrasonic transmitters attached to the lower jaw and eight ultrasonic receivers connected to the upper jaw through an arch. An extremely high accuracy of 50 micrometres can be achieved with this device [31].

Our study is the most comprehensive comparison to date, examining the accuracy of seven different techniques commonly used to determine centric relation (CR) in everyday practice. Among the seven techniques, five showed significant differences along at least one axis compared to the most reproducible and widely accepted reference point, which was the MIP [9, 12]. Neither the Dawson technique nor placing the tongue to the border of the hard and soft palate showed significant differences from the reference point along the three axes. Positioning the tongue at the palatal rugae, although not significantly different along the y-axis, exhibited the largest deviation along the x-axis, suggesting that the use of this method could result in capturing the mandible in a more protrusive position than desired. Mandibular retrusion by the patient, as well as backwards pressure on the chin, clearly resulted in a retrusive position of the lower jaw, which, despite being highly reproducible according to the literature, carries the risk of capturing the jaw in a posterior condylar position. The results obtained from a gothic arch tracer (the apex of the arrow and the adduction field) significantly differed from the reference point along at least one axis. This may be due to the translational movement of the condyle, which occurs even with minimal opening, resulting in a significantly anterior and downwards condylar position measurement. Accordingly, dentists need to consider and accept certain inaccuracies when using this method in patients without tooth loss.

A systematic review was published in 2021, in which a search was conducted on the MEDLINE, PubMed, Cochrane, and Google Scholar databases using the terms ‘Centric relation techniques’ AND/OR ‘Retruded mandibular position’ for the period between 1998 and 2019. Out of 958 articles, only 9 met the criteria defined for the study. Among these, five studies included edentulous participants, while four examined individuals with complete dentition. Based on these latter articles, the review concluded that the Dawson technique was the most accurate overall for the determination of CR in dentate patients [32]. This finding is consistent with the results of the present study.

## Conclusion

According to this study, we can conclude that when comparing different methods used to determine the CR in patients with stable MIPs, the smallest deviation from the reference position was found with the bimanual mandibular manipulation technique derived from Dawson. The second smallest deviation was observed when the tongue tip was placed on the border of the hard and soft palate according to the linguomandibular homotrophy theory. Among the seven methods examined, five showed significant deviations compared to the MIP on at least one axis, but the magnitude of these differences was within +/- 1 mm. Therefore, methods that do not result in posterior deviation (apex of the gothic arch tracer, adduction field, Dawson technique, tongue at the palatal rugae, tongue at the hard-soft palate border) are likely to be applicable in clinical practice. Active and passive retrusion of the mandible both resulted in a more posterior position compared to the MIP, which was calibrated at the beginning of the examination.

## Data Availability

The datasets used and/or analysed during the current study are available from the corresponding author upon reasonable request.

## Abbreviations

CR: Centric relation
CBCT: Cone beam computed tomography
CI: Confidence interval
EPA: Electronic position analysis
GPT: Glossary of Prosthodontic Terms
MRI: Magnetic resonance imaging
MIP: Maximal intercuspal position
TMD: Temporomandibular disorders
TMJ: Temporomandibular joint
